# Music in palliative care: a qualitative study with patients suffering from cancer

**DOI:** 10.1186/s12904-019-0461-2

**Published:** 2019-10-07

**Authors:** Sandrine Pommeret, Jan Chrusciel, Catherine Verlaine, Marilene Filbet, Colombe Tricou, Stephane Sanchez, Louise Hannetel

**Affiliations:** 1 Palliative Care Unit, Centre Hospitalier Troyes, Troyes, France; 2 Department of Medical Information Evaluation and Performance, Centre Hospitalier Troyes, 101 Avenue Anatole France, 10003 Troyes, France; 3 Department of Supportive and Palliative Care, Centre Hospitalier Lyon-Sud, Lyon, France

**Keywords:** Palliative care, Music, Qualitative study, End-of-life care

## Abstract

**Background:**

The palliative care unit is an emotionally challenging place where patients and their families may feel at loss. Art can allow the expression of complex feelings. We aimed to examine how cancer patients hospitalized in the palliative care unit experienced a musical intervention.

**Methods:**

We conducted a qualitative study based on semi-structured interviews. The study took place in a palliative care unit from 18 January 2017 to 17 May 2017. Two artists performed in the palliative care unit once a week from 9:30 am to 5:30 pm. The data from patient interviews were analysed based on an inductive approach to the verbatim accounts.

**Results:**

The accounts we gathered led us to weigh the positive emotions engendered by this musical intervention against the potential difficulties encountered. The artists opened a parenthesis in the care process and brought joy and well-being to the palliative care unit. Patients also encountered difficulties during the intervention: reference to an altered general state, to loss of autonomy**;** a sense of the effort required, of fatigue; an adaptation period; reference to the end of life, to death; a difficulty in choosing songs.

**Conclusions:**

Although music appeared to benefit the patients, it sometimes reminded them of their altered state. The difficulties experienced by patients during the experience were also related to physical exhaustion. Additional studies are needed to determine the benefits of music for patients and their families in the palliative care unit.

**Supplementary information:**

**Supplementary information** accompanies this paper at 10.1186/s12904-019-0461-2.

## Background

End of life situations are accompanied by psychological, emotional and social changes. Palliative care practitioners should adopt a patient-centered approach to address the complex needs of their patient [1]. The therapeutic effects of art in the context of palliative care have received increasing attention in recent years. A survey study of 300 hospices in the United States showed that music therapy is the second most common complementary therapy [2, 3]. A randomized study conducted by Hilliard showed improved quality of life measured on the scale of the Hospice Quality of Life Index-Revised (HQOLI-R) among 80 patients treated at home for terminal stage cancer following music therapy [4]. A meta-analysis by Bradt et al. reports that music may reduce anxiety, pain, fatigue and improve quality of life in cancer patients, although the level of evidence was low in the research considered in the study [5]. A pilot study conducted in Japan used salivary cortisol to show decreased stress levels after a musical intervention in the hospice [6]. The effects of music therapy are often reported in the form of direct accounts, as in the patient narratives gathered by music therapist Claire Oppert [7]. These accounts can complement evidence from quantitative studies by helping explain the mechanisms involved in the process, thus refining our understanding of the effects of the intervention [8]. They unravel existential and psychological aspects of the patient [9]. Reminiscence of various memories could play a role in the effectiveness of musical interventions [10]. Music therapy techniques may be classified as active (where patients participate in music creation) or receptive [11]. Live music has been shown to elicit more favorable responses than recorded music in cancer patients [12]. Our palliative care department hosts two artists each week. They are not music therapists, but their participation is more than musical entertainment since the goal is not to distract patients but rather to open a range of possibilities beyond care. The goal of this qualitative study was to examine how patients hospitalized in the palliative care unit experienced a musical intervention.

## Methods

### Context of the intervention

#### Setting

Our palliative care unit has 14 rooms. The average length of stay in 2016 was 13.1 days. The study was conducted from 18 January 2017 to 17 May 2017.

#### Intervention

The artists signed a confidentiality agreement and a contract with the hospital. They were present in the palliative care unit in pairs once a week, on Fridays, from 9:30 am to 5:30 pm. At least one of the two members of the pair was present the following week to ensure continuity in the intervention. The intervention consisted of the artists singing and playing a musical instrument (guitar, piano, percussion instruments). The artists’ presence was announced in advance to patients and families. They began playing in the hallway for 30 min and then joined the medical team for a briefing. A member of the team always introduced the artists. They could play during medical care, lunchtime, family visits or at other times if convenient. The care staff and artists interacted, complemented and supported each other, and were sometimes present in patients’ rooms at the same time, depending on their workload, the desire of patients and their families, and the events of the day. For some patients, the time spent with the artists was private, whereas for others it was shared with family and/or staff; for others still, it took place in the hallway. A debriefing occurred with the team at the end of each day. The artists had a dress code making them easy to identify. A debriefing was organized with the palliative care team at the end of the day.

### Patient population

#### Inclusion criteria

- Patients receiving palliative treatment for cancer in the palliative care department who agreed to meet the artists during the study period

#### Exclusion criteria

- Patients unable to communicate

### Methodology

This was a qualitative study based on semi-structured interviews using Interpretative Phenomenological Analysis [13], a method aiming to uncover the meaning of human experience through the identification of themes in their discourse. The interviewer was a female clinical psychologist working in the palliative care unit. Some of the patients had consulted her before the study. The study was presented to patients as research conducted to obtain an academic degree. Consecutive patients were approached for audio-recorded face-to-face interviews which took place in the palliative care unit. The interviewer was alone with the participants during the interviews. The interviews were transcribed, and field notes were taken during the interviews. The questions guiding the interviewer are shown in Additional file 1. The data were vertically analysed based on an inductive approach to the verbatim accounts. Themes were derived from the data. Cross-sectional analysis was jointly conducted with a doctor from the palliative care unit. The COnsolidated criteria for REporting Qualitative research (COREQ) analytical framework [14] was used. The interview grid for this study was tested and did not require any modifications. The patients in the test phase were as such included in the study. Dual coding was conducted by the psychologist and one of the doctors from the palliative care unit. Only patients receptive to music and who agreed to meet with the artists were interviewed. The interviews were conducted on the same day as the intervention, except for one patient (Table 1).

**Table 1:** Description of the study population

Patient	Age	Personal situation	Reason for hospitalization/Pathology	Duration of interview	Time between intervention and interview (days)
P1	80–90	Widow	Mammary neoplasia, end-of-life care	15’	0
P2	50–60	Divorced	Recurrent lung cancer treated by radiotherapy	13′07	0
P3	60–70	Common law	Multiple metastatic melanoma under hormone therapy, end-of-life care	20′02	0
P4	80–90	Married	Prostate cancer with bone metastasis, nausea	24’	0
P5	70–80	Married	Metastatic adenocarcinoma of the right breast, fatigue	12′14	0
P6	60–70	Single	Bladder neoplasm, end-of-life care	11′31	0
P7	40–50	Common law	Squamous cell carcinoma of the cervix, metastatic, end-of-life care	18′16	2
P8	70–80	Married	Progressive brain tumour, fatigue, dysphagia	38′56	0
P9	60–70	Single	Pulmonary adenocarcinoma, chest pain	14′40	0
P10	70–80	Married	Probable metastatic breast cancer, end-of-life care	19′38	0

## Results

Ten patients were interviewed (Table 1). One interview was carried out for each patient. The mean age was 69, with a range (rounded to the nearest decade) from 50 to 90. All patients except one were interviewed the same day as the intervention. Numerous patients were excluded because they could not communicate. One patient among those who could have been interviewed refused the interview. One interview was stopped because the patient was tired. The interviews lasted from 11 to 39 min. Saturation was reached with the ninth interview.

## Positive aspects

### A source of satisfaction and wellbeing

The majority of patients mentioned positive and pleasant emotions such as wellbeing, relaxation and joy: *“I felt happy and good … Yes, that’s it” (P1): “It really is heart-warming, isn’t it?” (P4).* Most of the patients commented on the quality of the performance: *“and they really sang very, very well” (P3); “Yes, it was not bad, for two people … it flowed nicely. Yeah, one was really good … it was well adapted, it was well rehearsed already” (P9).* Some people expressed the fact that they were moved by the songs performed: *“Oh … I got emotional (voice trembling, brings her hand to her throat), I didn’t want to cry in front of them because I’m emotional... I swear, I wanted to shed a tear” (P3)*; whereas others were confronted with the challenge of expressing their feelings in words: *“Um … you can’t even imagine it” (P1); “It was strange … it was good” (P9).*

Other patients conjured images suggesting envelopment and immersion: *“A cocoon (accompanied her words with an enveloping movement with her hand)” (P8); “Right now, I’m in it, I’m right in the midst of it” (P2).* In several cases, patients’ emotions were released after the fact, during the interviews: *“Um (silence) nothing but pleasure (voice trembling)” (P4).* The atmosphere during most of the interviews was quite upbeat and relaxed, punctuated with laughter (we recorded 23 patient laughs). The patients expressed humour, despite the emotional load at certain times: *“Plus, they were charming, they were young, they were beautiful, they smelled like young blood (laughs)” (P8); “Three by Claude François, because they couldn’t remember the others (laughs)” (P1).* Most of the patients felt the need to appear active and/or tried their best to participate in the songs with the artists: *“Well, I even sang along with them” (P2); “She said to me: shall I let them in? And I said oh yes, I want to participate!” (P8).*

### A source of attention and comfort

Several patients were touched by the attention that the artists gave them, talking of their devotion or even their sacrifice: *“They sacrifice hours that they could spend outside to entertain us” (P1).* The patients interviewed recognized that the experience allowed them to compensate for a lack of visitors: *“Oh … the timing was perfect … because … since I knew my sister wasn’t coming...so … I didn’t have anyone coming...I was going to be...all alone...aside from the TV...but maybe that’s not as good” (P2).* It even encouraged some patients to want to leave their rooms: *“Because there are other things too, there are not only … there are exhibits, all that, so … it’s … good, there are singers and then there are other things that maybe I could also go and see …*” *(P7).*

Some patients saw the performance as an antidote for boredom: *“it helps pass the time” (P6).* A sense of hope for an improvement in health or even a cure, of regaining autonomy was expressed in most of the comments: *“I mean it allows people to say there you go, maybe you’ll be able to make it through this after all!” (P5).*

### A vector for social ties and sharing

For several patients, being able to offer and share this musical experience with at least one family member was important. They enjoyed being able to please others: *“I enjoyed it for all the people who were present around me, yes” (P4).* Patients also mentioned how much they enjoyed sharing this artistic interlude with the care staff and they commented on the friendly atmosphere it created: *“plus there were back-up singers (several staff members: a nurse, auxiliary and administrative manager), they were good (laughs), no but it’s true, they were really very good and the little group that was there, that everyone was participating and singing and everything” (P3); “and real upbeat because … even some of the staff was participating and all” (P7).* During the interviews, people sometimes expressed concern for other patients in the unit by highlighting a sense of empathy or belonging: *“It maybe also lets people who do not have a lot of visitors, who do not have a lot of family, all that, to have someone come to see them even if it’s just to sing, even if it’s just for the music. It allows them to not be isolated, to … to, maybe to have the impression that someone is nevertheless coming to see them” (P7).*

### A catalyst for memories

Many people highlighted the family memories that the experience awoke in their minds: *“It reminds me of memories … It also reminds me of when I would go see him at the Olympia Hall” (P3).* A few patients went so far as to recognize that this artistic encounter had created new memories, for the future: *“Yes, he filmed it and since the children from [town] are coming tonight, they’re coming to the house, so that way he will be able to show my daughter” (P3); “they are nice memories [ …]* *Yes, for me they’re memories” (P8).*

## Limits and difficulties

### Reference to an altered general state, to loss of autonomy

For many of the patients, the interaction with the artists also made them reflect on their gradual loss of autonomy and their altered state of health: *“No, it’s no longer me, I was not like this (silence). I was effervescent” (P8); “the thing is, I need to be fixed back up so that I could sing well with them” (P2).*

### A sense of the effort required, of fatigue

Hosting the artists was sometimes costly in terms of effort and fatigue: *“because when you’re suffering at the same time, it’s hard to be fully there with them …*” *(P1); “.....I’ll try my best” (P2)”; “A bit tired, just a little …*. *well, I’m not sure if that’s why” (P4).*

### An adaptation period

Several patients were hesitant when the project was presented and pointed out the need for an adaptation period:

*“because yesterday, they spoke to me about it, the doctor, um...the nurses, they told me about it...and then um...at the time...no, I wasn’t really interested...honestly, it’s true at the time...but now, honestly, I enjoyed it” (P3).* The only patient that did not need time to accommodate for the intervention was P8, however the project had been presented to the patient before admission to the palliative care unit *“I told them they could come at my place, (referring to the room) this is my place” (P8).*

### Reference to the end of life, to death

A few patients mentioned and raised questions about the end of their life and/or their death. This could generate anxiety: *“And I said that this was the last time I’d see it … yeah … (silence)” (P3).*

### Difficulty choosing songs

Patients sometimes acknowledged having trouble: “*I was a bit uncomfortable not knowing which songs to ask for” (P7),* “*We were a bit surprised, sometimes, you know, we were there and ... they asked us, they asked us, and then... I know all the songs but then...well, erm, I couldn’t remember, I couldn’t recall which one it was” (P4).* Only one person said that they were easily able to spontaneously choose their songs: *“Yes, I told them, you can prepare this and this and this!” (P8).*

### A pre-existing taste for music

It should be noted that most of the patients already enjoyed listening to music or singing in their everyday lives, outside the hospital and, as such, they were favourably disposed to welcome the artists: *“Personally, I like music, I’m cultural: I like the cinema, theatre, music, dance” (P8); “I really like singing like this because at home I play, I mean I used to play, my stereo very, very loud. Martine would sing at the top of her lungs (laughs) and she danced” (P3); “I love the guitar” (P7).*

## Discussion

The accounts we gathered led us to weigh the positive emotions engendered by this musical intervention against the potential difficulties encountered. The themes that emerged from our study differed from other similar studies [15] by the addition of concepts related to the shortcomings of music therapy. Although music appeared to benefit the patients, sometimes it reminded them of their altered state. The difficulties experienced by patients during the experience were also related to physical exhaustion. Clinical psychologist and music therapist Edith Lecourt [16] writes that “music can provide a restful space when it acts as a container, but it can also be a sphere for pleasure and play” (translated here). This sentence is a good illustration of the statements made by the patients interviewed. For many, their emotions were drawn from the register of pleasure, relaxation and contentment.

Sometimes, what was felt remained in a raw state, below the level of words. Music can be felt more than it can be explained: some of the patients interviewed indeed used sensory terms or terms connected to movement to express their experience. Their testimonies remind us of the “psycho-physiological change” described by Schmid et al. [1]. Several patients referred in their own manner to a feeling of envelopment, immersion, to express their wellbeing. For Edith Lecourt [16], a good share of musical pleasure is indeed tied to this ability to become attuned with others.

The artists in our study adjusted to each other and also adjusted to the patients and family members present. Such adjustment is one of the foundational tenets of analytical music therapy. For many of the patients interviewed, sharing this musical experience with family or care staff turned the occasion into a moment of joy. For a short time, their room became a place of interaction, a playful space. The inclusion of care staff and family reinforced a sense of belonging and safety in patients, helped overcome their sentiment of solitude. This finding can be contrasted with Bieligmeyer et al. [17], where a vibroacoustic musical therapy intervention using a sound-bed did not alter social extraversion scores of cancer patients. This suggests that the effects of musical interventions can be as diverse as the interventions themselves. Likewise, Peng mentions that each encounter in her study of music in the palliative care setting was idiosyncratic [15].

A good number of patients recalled with pleasure details from their personal and cultural history after the performance. The songs and music worked like “acoustic reminders of childhood memories” and encouraged patients to open up, to travel back and forth between the past, the present and the future. Musical interventions with patients in end of life situations allow them to take stock of their lives, to share anecdotes with others. The artists as such become witnesses to patients’ “path of effort to restore the self” in a period of existential crisis (an expression from Jacquemin [18], translated here). For a moment, their identities are reframed from patients to “people with unique pasts, interests and personalities” [8]. This theme resembles the “reflections” cluster in Peng’s study of music in palliative care [15]. Themes related to spirituality were less developed in our study, perhaps for cultural reasons [19].

During the interviews, many of the patients mentioned the renunciation and mourning work they still needed to accomplish regarding their illness and their loss of autonomy in connection with the resurgence of memories provoked by the songs. Sometimes, the topic of death was also present. However, the interaction with the artists filled them with hope for improvement. This psychological upsurge [20], was also found in the desire of patients to participate in singing and to make plans for the future.

One possible limitation of our study is the small number of participants. Further studies could include more centres to search for other themes. The investigator tried to minimize interaction with patients prior to their interview as much as possible, but she was nevertheless the unit psychologist, which increases the “desirability bias” always present in this type of study.

## Conclusions

This qualitative study examined the benefits that the introduction of music and song can have on hospitalized patients receiving palliative care.

The results suggest that this living art form has a role to play in care facilities, that it fosters dialogue and calls on patients – who often see themselves primarily as objects of care – as people, as individuals. Music can help to humanize relationships in the palliative care setting. This finding was consistent throughout the patient narratives in the study. Flusser has argued that the interaction with artists allows “dialogue between subjects that creates a sphere of freedom in care-based relationships, creating pleasure and desire, fuelling an energy to become, a desire to live” (translated here) [21]. However, music can elicit negative reactions from patients due to fatigue or by provoking thoughts about their disease and loss of autonomy.

It is important for music therapists not to work alone, to have sufficient resources to tackle situations which are sometimes disconcerting [22] – there are indeed many reasons why it is necessary to include the living arts in the care project of a unit, but the project needs to be backed by a trained, multidisciplinary team.

Further research could include interviews of the families of patients in the palliative care unit regarding the benefits they derived from the intervention [23]. The involvement of artists could help families see the patient no longer through the lens of someone who is dying, but rather as a person living out the end of their life. It could as such help with the attachment/detachment process. Remembering these moments could later help families with the mourning process [24].

## Supplementary information

**Additional file 1. ** Questions for the semi-structured interview. This file contains the questionnaire which was developed specifically for this study.

## Data Availability

The datasets analyzed during the current study are available from the corresponding author on reasonable request.

## Abbreviations

COREQ: COnsolidated criteria for REporting Qualitative research
HQOLI-R: Hospice Quality of Life Index-Revised
