# Supplementary material for: Music in palliative care: a qualitative study with patients suffering from cancer
Source: BMC Palliat Care. 2019 Oct 7;18:78. doi: 10.1186/s12904-019-0461-2 (PMC6781358; doi:10.1186/s12904-019-0461-2)
Supplement: Supplementary file 1 — Additional file 1. Questions for the semi-structured interview. This file contains the questionnaire which was developed specifically for this study. [file 12904_2019_461_MOESM1_ESM.pdf]

Questions for the semi-structured interview

Order	Question
1	Tell me about your meeting with the artists, including the circumstances. Was it the first time you met them here?
2	How did you feel about their proposal to sing for you? What did you think about the choice of songs?
3	What happened to you and your entourage at this moment? How did you feel at this time?
4	How did you feel afterwards? What did you retain from this encounter? What effect did it have on you?
5	What will you remember from this experience? Say what comes to your mind now, without thinking about it.
6	What can we do to make the intervention better?
7	Is there anything else you would like to mention?
